# Effectiveness of a “5E+AI” teaching model on science communication in medical students: a contribution to global health literacy

**DOI:** 10.3389/fpubh.2026.1916714

**Published:** 2026-08-07

**Authors:** Huiru Dai, Minling Liu, Tingwei Li, Jiancheng Wang, Shuo Fang

**Affiliations:** 1 Department of Oncology, The Seventh Affiliated Hospital of Sun Yat-sen University, Shenzhen, Guangdong, China; 2 Department of Education, The Seventh Affiliated Hospital of Sun Yat-sen University, Shenzhen, Guangdong, China

**Keywords:** 5E teaching model, artificial intelligence, health literacy, medical education, medical science communication education

## Abstract

**Background:**

The improvement of global health literacy depends on health professionals having effective public science communication capabilities. However, worldwide, medical education systems still largely lack training in science communication for medical students. How to construct a systematic and scalable model for cultivating scientific communication capabilities is a key issue currently faced by the reform of medical education.

**Objective:**

Our study aims to evaluate the effect of the “5E+AI” teaching model on the cultivation of medical students' science communication capabilities, and to assess the impact of this model on students' learning participation, acquisition of medical knowledge, and creation of popular science works.

**Methods:**

A historical controlled quasi-experimental design was employed. Twenty-nine medical students enrolled in the “Fundamentals and Practice of Medical Science Popularization” course at Sun Yat-sen University during the 2025-1 semester served as the historical control group (receiving lecture-based, instructor-centered teaching with PowerPoint presentations and group discussions, but without AI-assisted tools), while 28 students enrolled in the same course during the 2025-2 semester served as the experimental group (receiving the “5E+AI” teaching model). The two groups are the same in terms of teaching content, total class hours, teachers, teaching materials, assessment methods, etc. The evaluation indicators include participation score, medical knowledge test score, final score of the science communication project and student self-report questionnaire. Intergroup comparisons were performed using the Mann–Whitney *U*-test.

**Results:**

The experimental group scored significantly higher than the control group on all three primary outcome measures: participation scores (median 85.00 vs. 80.00, *U* = 220.00, *P* = 0.002), medical knowledge test scores (median 100.00 vs. 80.00, *U* = 210.00, *P* = 0.001), and science communication project scores (median 85.00 vs. 85.00, *U* = 213.00, *P* = 0.001). In addition, the experimental group reported significantly higher scores than the control group across all three dimensions of the self-report questionnaire (*P* < 0.05).

**Conclusions:**

The “5E+AI” teaching model helps enhance medical students' classroom participation, ability to acquire medical knowledge and science communication skills. In this teaching model, artificial intelligence can help students address the issues of knowledge deficiency and resource constraints. Meanwhile, the 5E teaching framework provides students with structured teaching scenarios. Together, they enhance teaching effectiveness and offer replicable and adaptable local solutions for the reform of medical communication education in the context of global health literacy promotion.

## 1 Introduction

Health literacy is a strong predictor of population health outcomes. Individuals with limited health literacy tend to make riskier health decisions, engage in more harmful behaviors, experience higher hospital readmission rates, and have poorer overall health. On the other hand, stronger health literacy has been linked to better clinical decision-making, improved health status, narrower health disparities, and potential cost savings of roughly 8% on total healthcare expenditures (1).

Healthcare providers occupy a unique position as both primary deliverers of clinical services and trusted sources of health information for the public. Their ability to communicate medical knowledge effectively thus has a direct bearing on community health literacy levels (2, 3). Recognizing this, the American Association for the Advancement of Science (AAAS) and other international bodies have repeatedly urged medical schools to include science communication in their curricula (4, 5). In practice, however, most medical programs still lack formal requirements in this domain. Training in public communication and interpersonal health dialogue remains sparse, leaving many students ill-equipped to translate their expertise for lay audiences. This gap in training not only hinders students' ability to communicate medical knowledge to the public but may also limit broader societal gains in health literacy.

Efforts to teach science communication to medical students have emerged at several institutions, but they differ considerably in format and intensity. At Indiana University, training is delivered through short workshops (6); at Edinburgh, students engage in a longer course that includes both theory and hands-on practice. Despite these examples, the field lacks a shared framework. Curricula are frequently improvised, teaching methods are inconsistent, and digital tools are rarely integrated effectively. Designing a training model that is both practical and scalable—and that links directly to health literacy —remains an open challenge.

Medical science communication places heavy cognitive demands on learners. Students must simultaneously master clinical content, assess the prior knowledge of diverse audiences, and translate specialized terminology into accessible language. For educators, this raises the challenge of designing instructional approaches that reduce these cognitive burdens while enabling the gradual acquisition of communication skills. The 5E model (7), originally developed by the Biological Sciences Curriculum Study in the 1980s (8, 9), offers a structured pathway for this purpose. Its five phases, Engage, Explore, Explain, Elaborate, and Evaluate, have provided a framework that organizes complex learning tasks into manageable stages, reducing cognitive load and supporting the incremental development of skills. The model has been widely applied in science and health education, with evidence demonstrating its effectiveness in structuring inquiry-based learning and supporting skill development in medical and health professions education (10, 11).

However, the 5E model, though structurally sound, faces two challenges in science communication education. First, its “Explore” and “Elaborate” phases demand intensive individualized guidance that instructors cannot easily provide in large classes with limited hours. Second, authentic communication practice requires repeated low-stakes interactions with diverse audiences, but traditional methods like community outreach or peer evaluation cannot feasibly deliver within a single semester. These gaps indicate that the 5E framework needs supplementary support to enable the high-frequency, personalized practice essential for mastering science communication.

Artificial intelligence is opening new avenues for science communication training. A 2024 scoping review examined the use of AI-driven virtual patients in healthcare education and found that such tools are increasingly viable for scalable communication practice (12). Other recent work has tested generative AI and conversational chatbots in medical training contexts, with promising results for patient-centered communication skills (13, 14). Specifically, these studies suggest that AI can address the two key challenges identified above: providing individualized feedback at scale and enabling low-stakes, repetitive practice with simulated audiences. Despite this momentum, few studies have explored how AI might be systematically integrated into a structured teaching framework like the 5E model for the specific purpose of science communication training. Most existing applications use AI as a standalone tool rather than embedding it within a pedagogical structure that guides the learning progression. To address this gap, we embedded AI tools into the 5E instructional sequence and evaluated their contribution to science communication competence. This paper reports the resulting course design, its implementation, and the outcomes observed.

## 2 Methods

### 2.1 Study design

This course is offered as an elective and draws limited enrollment each semester. These constraints made a randomized concurrent design unfeasible. We therefore adopted a historical controlled quasi-experimental design.

### 2.2 Participants

Participants were 57 clinical medicine undergraduates who enrolled in the “Fundamentals and Practice of Medical Science Popularization” course across two consecutive semesters (2025-1 and 2025-2). Students from the 2025-1 semester (*n* = 29) were assigned to the control group and received conventional instruction. Those from the 2025-2 semester (*n* = 28) formed the experimental group and were taught using the “5E+AI” model.

### 2.3 Teaching intervention

The course was delivered over 12 weeks, with one concentrated session per week. Each session comprised three modules: medical knowledge and science communication theory (two class hours) and practical project creation (approximately one class hour). Both groups were identical in course syllabus, total hours, instructor team, teaching materials, assessment formats, scoring rubrics, and evaluation criteria, scoring rubrics, differing only in pedagogical approach.

Although students were not restricted from accessing publicly available AI tools outside classroom activities, the control group did not receive any structured AI-assisted learning activities, including AI-generated content support, AI-based simulation practice, or personalized AI feedback. In contrast, AI was systematically integrated into the experimental group as a core component of the 5E+AI teaching model.

#### 2.3.1 Control group: conventional teaching model

(1) Teaching tools: PowerPoint-based lectures without AI-assisted tools. (2) Teaching methods: primarily lecture-based, supplemented by classroom questioning and group discussion. (3) Practical project creation: students completed science communication projects in small groups through independent literature review and group discussion, without AI-assisted content generation, AI-simulated interactive training, or AI-based personalized feedback. (4) After-class support: instructors responded to student inquiries via email or during break periods.

#### 2.3.2 Experimental group: “5E+AI” teaching model

**Figure 1:**
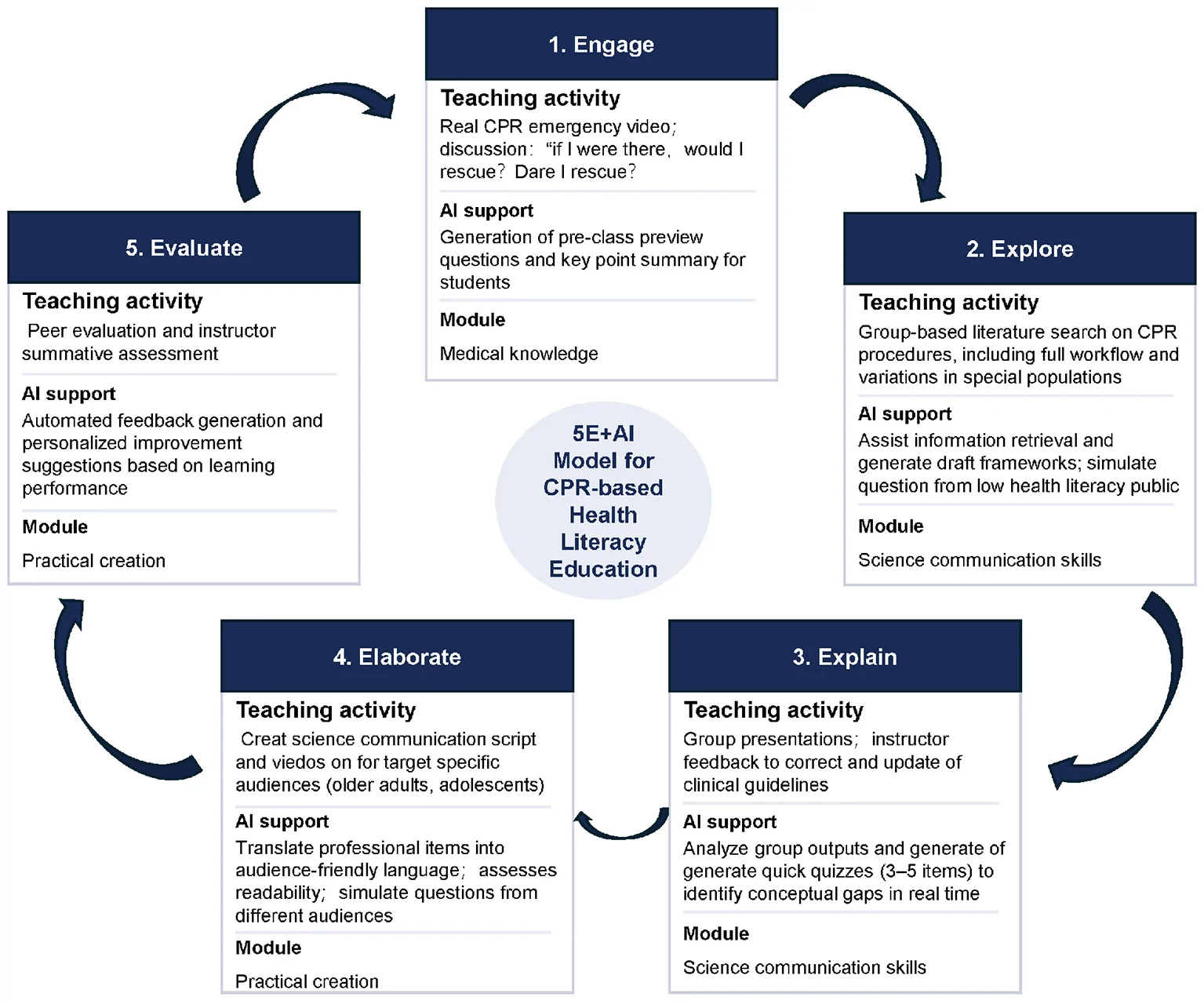
5E+AI instructional model for CPR-based health literacy education.

The experimental group adopted the 5E instructional model (Engage, Explore, Explain, Elaborate, Evaluate) as the organizational framework, with generative AI tools serving as assistive technologies covering three task types: content generation, simulation-based training, and feedback optimization. Within this model, AI was designed primarily to support students' learning process by providing on-demand information retrieval, simulated practice partners, and automated personalized feedback at each phase of the 5E cycle (Figure 1).

### 2.4 Outcome measures and research instruments

To evaluate the effectiveness of the intervention on science communication competence, we grounded our assessment framework in Lasswell's classic “5W” communication model (Who, Says What, In Which Channel, To Whom, With What Effect), a well-established framework that has been widely applied to structure the evaluation of science communication activities (15). Based on this framework, we selected three objective outcome measures and one subjective research instrument, each corresponding to specific elements of the communication process.

#### 2.4.1 Participation scores (outcome measure)

Participation Scores captured the communicator (“Who”) dimension—students' active engagement as science communicators in classroom discourse. We calculated participation scores based on three components: class attendance, in-class interaction, and group discussion performance. These scores ranged from 0 to 100 and served as a proxy for students' sustained engagement and routine performance across the course.

#### 2.4.2 Medical knowledge test scores (outcome measure)

Medical Knowledge Test Scores captured the message content (“Says What”) dimension—mastery of accurate clinical knowledge essential for effective health communication.

The knowledge test including lung cancer, influenza, hypertension, CPR, hepatitis B, Helicobacter pylori, and osteoporosis. It comprised 10 questions, with each question carrying 10 points.

#### 2.4.3 Science communication project scores (outcome measure)

Science Communication Project Scores captured the audience (“To Whom”), channel (“In Which Channel”), and effect (“With What Effect”) dimensions—the ability to design and deliver audience-appropriate, accessible, and engaging health messages.

Students completed science communication projects, which were then graded independently by faculty members with backgrounds in medical education. We took the average of their ratings as the final score for each project.

#### 2.4.4 Student self-report questionnaire (research instrument)

The Student Self-Report Questionnaire served as a supplementary research instrument to capture subjective perceptions that complement these objective measures.

The instrument contained 10 items rated on a 5-point Likert scale (1 = strongly disagree, 5 = strongly agree), organized into three dimensions: learning interest and motivation (item 1–2), self-perceived science communication competence (item 3–6), and course satisfaction (item 7–10).

The questionnaire was developed by the teaching team based on a review of relevant literature (16). To establish content validity, the initial item pool was reviewed by five experts in medical education and science communication, who evaluated the relevance, clarity, and comprehensiveness of each item. Based on their feedback, items were revised and the final version was determined. The item-level content validity index (I-CVI) ranged from 0.80 to 1.00. The scale-level content validity index (S-CVI) was 0.94, indicating excellent content validity. The scale demonstrated good internal consistency, with a Cronbach's α of 0.924. Students completed the questionnaire anonymously at the end of the semester. The response rate was 100% (29/29) for the control group and 93% (26/28) for the experimental group.

### 2.5 Statistical analysis

We conducted all statistical analyses using SPSS 26.0. The Shapiro-Wilk test revealed that score distributions and questionnaire responses in both groups departed significantly from normality (*P* < 0.05). Accordingly, we used the Mann–Whitney U test for between-group comparisons. To explore relationships among participation scores, medical knowledge test scores, project scores, and total questionnaire scores, we computed Spearman's rank correlation coefficients.

## 3 Results

### 3.1 Comparison between the two groups

#### 3.1.1 Participation scores

The experimental group demonstrated significantly higher participation scores [median = 85.00, interquartile range (IQR) = 80.00–90.00] compared to the control group [median = 80.00, IQR = 80.00–85.00] (*U* = 220.00, *P* = 0.002), indicating that students in the experimental group showed superior classroom engagement and daily performance (Table 1).

**Table 1:** Comparison of scores between the two groups.

Dimension	Control group (*n* = 29)	Experimental group (*n* = 28)	*U-*value	*P*-value
	Mdn (IQR)	Mdn (IQR)		
Participation scores	80 (80–85)	85 (80–90)	220.00	0.002^**^
Medical knowledge test	80 (70–90)	100 (90–100)	210.00	0.001^***^
Science communication project scores	85 (75–85)	85 (85–90)	213.00	0.001^***^

Data are presented as median (25th−75th percentiles). Mann–Whitney U-test was used for between-group comparison. ^**^P < 0.01, ^***^P < 0.001.

#### 3.1.2 Medical knowledge test scores

In the medical knowledge test, the experimental group's scores [median = 100.00, IQR = 90.00–100.00] were also significantly higher than those of the control group [median = 80.00, IQR = 70.00–90.00] (*U* = 210.00, *P* = 0.001; Table 1).

#### 3.1.3 Science communication project scores

Furthermore, the experimental group's science communication project scores [median = 85.00, IQR = 85.00–90.00] were significantly higher than those of the control group [median = 85.00, IQR = 75.00–85.00] (*U* = 213.00, *P* = 0.001; Table 1).

**Figure 2:**
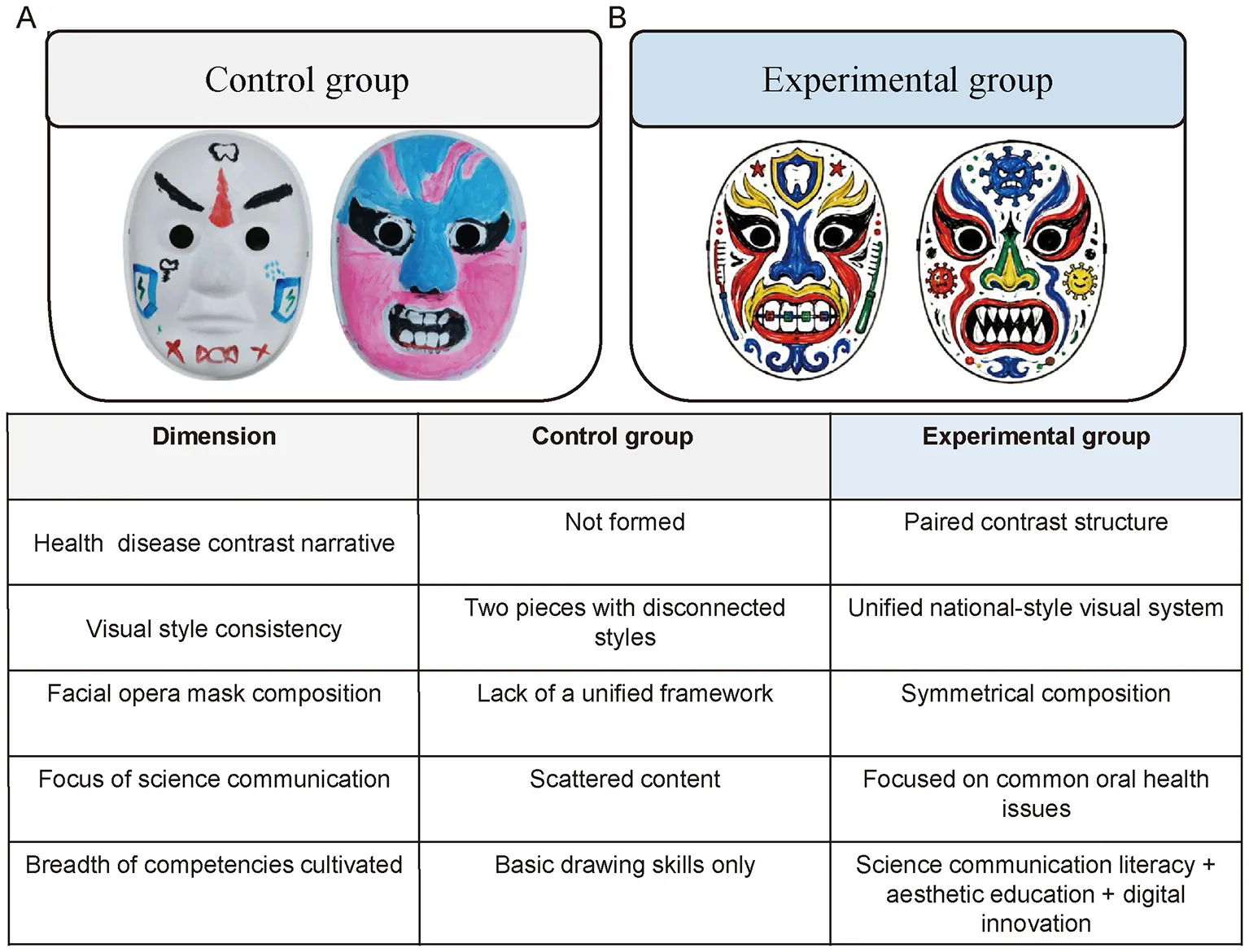
Comparison of student science communication works between the control group teaching group **(A)** and the experimental teaching group **(B)**.

In this part, we showed student project examples of two groups. We used facial masks as a medium for oral health science communication aimed at adolescent audiences. Figure 2A shows a representative project from the control group, and Figure 2B shows one from the experimental group. The control group's work appeared fragmented and lacked visual coherence. In contrast, the experimental group's project—completed with AI assistance—showed clearer narrative structure, better visual consistency, and stronger overall science communication quality (Figure 2).

#### 3.1.4 Self-report questionnaire scores

The questionnaire covered three dimensions: learning interest and motivation, self-perceived science communication competence, and course satisfaction. On all three, the experimental group outscored the control group (Table 2).

**Table 2:** Comparison of questionnaire dimension scores between the two groups.

Dimension	Control group (*n* = 29)	Experimental group (*n* = 26)	*U-*value	*P-*value
	Mdn (IQR)	Mdn (IQR)		
Total score	4.00 (3.80, 4.25)	4.60 (4.00, 5.00)	223.50	0.009^**^
Learning interest and motivation	4.00 (4.00, 4.50)	4.75 (4.00, 5.00)	241.00	0.012^*^
Self-assessed science communication competence	4.00 (3.50, 4.13)	4.25 (4.00, 5.00)	242.00	0.020^*^
Course satisfaction	4.00 (3.88, 4.75)	4.50 (4.00, 5.00)	231.50	0.012^**^

5-point Likert scale (1 = strongly disagree, 5 = strongly agree). ^*^P < 0.05, ^**^P < 0.01.

## 4 Discussion

Our results showed a clear advantage for the AI integrated cohort across all measured outcomes. The experimental group outperformed the historical control group on participation scores, medical knowledge tests, and science communication projects. We interpret this as preliminary evidence that the 5E+AI model not only supports knowledge acquisition but also strengthens the applied skills central to science communication. These findings also offer empirical support for integrating health literacy training into medical education, a direction the WHO has actively encouraged.

### 4.1 Synergistic effects on participation and engagement

A survey of 46 UK medical schools by Sarela et al. (17) found that non-academic factors, such as personal interest or cultural exposure, often outweighed academic considerations when students chose electives. This suggests that many medical students view elective courses more as an “experience” than as serious academic work. Given the competing demands of core clinical training, this mindset often translates into low engagement and passive participation. Our participation score data showed that the 5E+AI model improved both attendance and active involvement. Questionnaire responses confirmed that experimental group students reported stronger interest and motivation than their peers in the control group, a pattern consistent with earlier work (11). We attribute these gains largely to the synergistic interplay between the 5E framework and AI support. The 5E's five phases are organized around problem-oriented tasks and short cycle iterations. This design allows students to complete smaller, meaningful chunks of work in sequence, building a sense of progress and achievement along the way. That sense of accomplishment likely reinforced their willingness to stay engaged. Crucially, AI tools amplified this structural advantage by removing two key barriers to participation. Students no longer struggled to find relevant information during the Explore phase, as AI-assisted searching gave them quick access to what they needed. They also gained a safe space to practice their communication strategies during the Elaborate phase, as AI-simulated audiences allowed them to test their messages without real-world pressure. By removing these barriers, AI transformed the 5E structure into an accessible and engaging learning experience.

Self-determination theory (18) offers a useful lens for interpreting these patterns. The theory holds that when learners experience autonomy, competence, and relatedness, their intrinsic motivation and satisfaction rise together. In our setting, AI-supported exploration gave students greater autonomy in directing their learning, while the iterative feedback embedded within the progressive structure of the 5E phase helped build their sense of competence. However, we did not directly measure these psychological constructs, so this interpretation remains tentative and should be tested in future work.

### 4.2 Synergistic effects on medical knowledge acquisition

The experimental group's higher knowledge test scores suggest that the 5E+AI model enhanced content retention through complementary mechanisms. Traditional lecture-based instruction (19) places students in a passive receiving role, often resulting in superficial understanding and limited retention. The 5E model addresses this by redesigning the learning process (20). Rather than simply receiving information, students actively construct knowledge through exploration, explanation, and elaboration.

Within the limited contact hours of a single semester, however, the 5E structure alone could not provide the frequency and immediacy of feedback needed for rapid knowledge consolidation. AI integration may address this gap in several ways. When students had questions during exploration, AI provided immediate answers that prevented misconceptions from taking hold (21). During practice sessions, AI-generated Questions&Answers forced students to retrieve and apply what they had learned, strengthening their grasp of the material. And through personalized feedback, AI directed students back to the underlying clinical mechanisms they needed to review, helping them consolidate their understanding efficiently (22, 23). Together, these mechanisms intensified the learning that the 5E structure was designed to promote, enabling students to achieve better content mastery within the same timeframe.

In sum, the 5E framework provided the pedagogical structure for active learning, while AI supplied the feedback that made that learning stick within the semester timeframe.

### 4.3 Synergistic effects on science communication project quality

For undergraduate medical students, creating content for lay audiences is a cognitively demanding task requiring clinical knowledge, audience analysis, language translation, format selection, and visual design. The 5E framework addressed this by decomposing the project into manageable stages, allowing students to focus on one aspect at a time. This staged progression reduced cognitive load, lowered the barrier to entry, and made the overall task more approachable.

However, decomposition alone does not eliminate the practical difficulties students encounter at each stage. Even with a clear structure, students still struggle with where to find reliable information, whether their message is understandable to a real audience, and how to tell what needs improvement. AI addressed these difficulties in ways that differ fundamentally from its role in knowledge acquisition. Students saved time on preliminary work through AI assisted searching and drafting, freeing up energy for more thoughtful planning. Through simulated interactions, they could try out their messages and see how audiences might respond. And with personalized feedback, they could identify weaknesses and make targeted revisions—not just once, but throughout the development process (24, 25). Here, AI did not accelerate an existing learning process. It enabled students to overcome obstacles that would otherwise prevent them from producing high-quality work. The cumulative effect is visible in Figure 2: experimental group projects show clearer structure, stronger visual appeal, and greater audience awareness than control group work.

### 4.4 Implications for health literacy and medical education

At a broader level, these findings have implications for population health literacy and medical education reform. Health literacy depends not only on what the public knows but also on how well healthcare providers communicate. Traditional medical curricula have historically emphasized knowledge acquisition over public communication, leaving many students unprepared to engage lay audiences. The 5E+AI model offers a practical pathway for addressing this gap by embedding science communication training within a structured, scalable pedagogical framework. While our study was conducted at the course level and cannot claim direct effects on public health outcomes, it provides proof-of-concept evidence that structured teaching with AI support can build the communication skills that are essential for improving population health literacy.

### 4.5 Limitations

We also acknowledge several limitations. Because the course was offered only once per year, we could not recruit a concurrent control group. Therefore, a historical controlled quasi-experimental design was adopted, which may introduce potential confounding factors. In addition, students were aware of the instructional approach they received because the two cohorts were recruited from different semesters, and this awareness may have introduced expectation bias. Although the control group did not receive structured AI-assisted teaching activities, we could not completely exclude the possibility that students independently accessed publicly available AI tools outside the course. Furthermore, the sample size was modest, the study was conducted at a single institution, and all assessments were performed immediately after the course, limiting the generalizability and long-term interpretation of our findings. Future studies should include larger multi-center samples, concurrent randomized controlled designs, longer follow-up periods, and systematic monitoring of AI usage behaviors to further validate the effectiveness and broader applicability of the model.

## 5 Conclusions

We developed and tested a structured teaching model that integrates AI tools into the 5E instructional framework for medical science communication education. In this elective course, the experimental group outperformed the control group on measures of knowledge, project quality, and course evaluation. The combined approach—structured instructional design plus AI support—appeared to enhance the learning experience and may have contributed to better outcomes. We propose this as a potentially scalable model for other institutions seeking to strengthen science communication training in medical curricula. Further research, ideally through multi-center randomized controlled trials, is needed to confirm its long-term effectiveness and transferability.

## Data Availability

The original contributions presented in the study are included in the article/supplementary material, further inquiries can be directed to the corresponding authors.
